# National recommendations of the Croatian Chamber of Medical Biochemists and Working group for Laboratory hematology of the Croatian Society of Medical Biochemistry and Laboratory Medicine: Management of samples with suspected EDTA-induced pseudothrombocytopenia

**DOI:** 10.11613/BM.2024.030504

**Published:** 2024-10-15

**Authors:** Lara Milevoj Kopčinović, Gordana Juričić, Dragana Antončić, Fran Smaić, Brankica Šimac, Ivana Lapić, Vanja Radišić Biljak

**Affiliations:** 1 Department of Clinical Chemistry, Sestre milosrdnice University Hospital Center, Zagreb, Croatia; 2 School of Medicine, Catholic University of Croatia, Zagreb, Croatia; 3 Department of Laboratory Diagnostics, General Hospital Pula, Pula, Croatia; 4 Clinical Department of Laboratory Diagnostics, Rijeka Clinical Hospital Centre, Rijeka, Croatia; 5 Department of Laboratory Diagnostics, General Hospital Dr. Josip Benčević, Slavonski Brod, Croatia; 6 Clinical Department of Laboratory Diagnostics, University Hospital Dubrava, Zagreb, Croatia; 7 Department of Laboratory Diagnostics, University Hospital Centre Zagreb, Zagreb, Croatia; 8 University of Zagreb, Faculty of Pharmacy and Biochemistry, Zagreb, Croatia; 9 Department of Medical Laboratory Diagnostics, University Hospital Sveti Duh, Zagreb, Croatia; 10 Department of Sport and Exercise Medicine, University of Zagreb, Faculty of Kinesiology, Zagreb, Croatia

**Keywords:** thrombocytopenia, pseudothrombocytopenia, hematology analyzers, procedures, harmonisation

## Abstract

Pseudothrombocytopenia (PTCP) is defined by the occurence of spouriously low platelet count as a consequence of *in vitro* platelet aggregation. It is a rare and benign artifact, not associated with any specific disorder or therapy, that becomes clinically relevant when it is not timely and reliably recognized. Thus, it may result in inappropriate clinical decisions (*i.e.* unnecessary further testing, misdiagnoses and potential patients’ mismanagement) unavoidably compromising patient safety. The most common form of PTCP is caused by ethylenediaminetetraacetic acid (EDTA). Several approaches for the management of samples with EDTA-induced PTCP have been described in the literature. However, expert recommendations are scarce. The scope of these recommendations is to assist in achieving national harmonisation in laboratory management (*i.e.* detecting and reporting platelet counts) of samples with EDTA-induced PTCP. These minimal recommendations were prepared by the members of the joint working group of the Croatian Chamber of Medical Biochemists and Working group for Laboratory Hematology of the Croatian Society of Medical Biochemistry and Laboratory Medicine, and might be customized according to specific conditions (*i.e.* personnel and equipment) of each individual laboratory. These recommendations are primarily intended to all laboratory professionals involved in the management of samples with EDTA-induced PTCP, but also to other healthcare professionals involved in collecting samples and interpreting complete blood count results.

## Graphical abstract

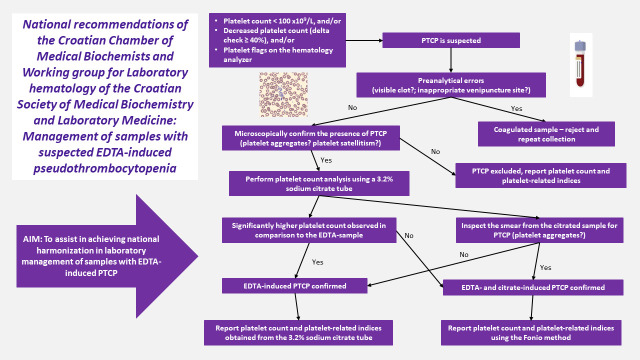

## Highlights

• National recommendations aim to harmonize the management of EDTA-induced pseudothrombocytopenia

• The influence of preanalytical errors should be excluded

• The presence of platelet clumps should be confirmed by peripheral blood smear inspection

• Platelet counts should be determined from the 3.2% sodium citrate sample

• If EDTA-induced pseudothrombocytopenia is confirmed, platelet counts are reported with appropriate comments

## Introduction

Thrombocytopenia is a common condition characterized by low blood platelet count (< 150 x10^9^/L) and associated with various disorders (*1*). Unlike thrombocytopenia, pseudothrombocytopenia (PTCP) is defined by the occurrence of falsely low platelet count as a consequence of *in vitro* platelet aggregation (*2*). This benign and rare phenomenon, presents in 0.03-0.27% of the general population and in up to 15.3% of patients with thrombocytopenia, and is not associated with any specific disorder or therapy (*2*). If not timely and reliably recognized, PTCP might result in unwanted clinical implications. The omitted recognition of this spurious cause of thrombocytopenia inevitably leads to inappropriate clinical decisions (*i.e.* unnecessary further testing, misdiagnoses and potential patients’ mismanagement), unavoidably compromising patient safety (*2*, *3*).

The most common form of PTCP is caused by the anticoagulant ethylenediaminetetraacetic acid (EDTA). Several approaches for the management of samples with EDTA-induced PTCP have been described in the literature. The addition of different compounds (*e.g.* amikacin or kanamycin) to the EDTA-sample with confirmed PTCP in order to prevent the formation of platelet aggregates or disaggregate platelets within already existing aggregates, rapid EDTA-sample analysis, collection and analysis of the EDTA-sample at 37 ^o^C, and use of alternative anticoagulants (*e.g.* acid citrate dextrose, magnesium sulfate or sodium citrate) have been suggested (*2*-*4*). Additionally, the potential usefulness of EDTA-sample vortexing for the purpose of disaggregating platelet clumps has also been investigated, but there is lack of objective evidence supporting the claim of its effectiveness for the complete disaggregation of platelet clumps and consequently accurate platelet counts determination in samples with PTCP (*5*-*7*). However, expert recommendations dedicated to the management of EDTA-induced PTCP are lacking.

Considering the heterogeneity of approaches available for the management of EDTA-induced PTCP, the scope of this document is to assist in achieving national harmonisation in laboratory management (*i.e.* detecting and reporting platelet counts) of samples with suspected EDTA-induced PTCP. The proposed minimal recommendations were prepared by professional consensus of the members of the joint working group of the Croatian Chamber of Medical Biochemists and Working group for Laboratory Hematology of the Croatian Society of Medical Biochemistry and Laboratory Medicine, and might be customized according to specific conditions (*i.e.* personnel and equipment) of each individual laboratory. These recommendations are primarily intended to all laboratory professionals involved in the management of samples with EDTA-induced PTCP. Furthermore, they are also intended to other healthcare professionals involved in collecting samples and interpreting complete blood count results.

## Recommended criteria for raising suspicion on PTCP

If at least one criterion is met, the presence of PTCP should be suspected and the recommended laboratory procedure for the detection and management of PTCP should be undertaken.

**Figure 1:**
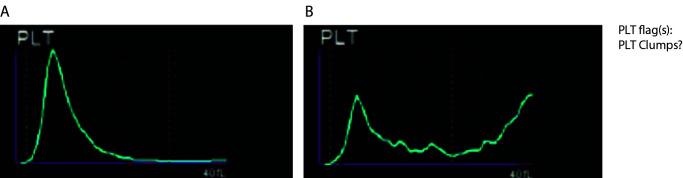
Comparison of platelet histograms obtained by the impedance method on the Sysmex XN-1000 hematology analyzer (Sysmex, Kobe, Japan): in (A) blood sample without and (B) blood sample with suspected PTCP. Adapted from (*11*). PTCP - pseudothrombocytopenia. PLT - platelets.

1. Low platelet count, *i.e.* < 100 x10^9^/L, determined on the hematology analyzer in a patient without any clinical signs or history of thrombocytopenia or platelet disorders, and without previous platelet count result; and/or
2. A significant decrease in platelet count (delta check ≥ 40%) compared to the previous patient result; and/or
3. The presence of platelet and/or leukocyte-associated flags on the hematology analyzer in conjunction with an altered platelet histogram (*2*, *3*, *8*, *9*) (Figure 1).

Pseudothrombocytopenia is influenced by the presence of immune (*i.e.* platelet autoantibodies), chemical (*i.e.* anticoagulants) and physical (*i.e.* time, temperature) factors which altogether contribute to the occurrence of this *in vitro* phenomenon. In the literature, PTCP is most commonly associated with the presence of autoantibodies cross-reacting with the glycoprotein IIb/IIIa complex (*i.e.* fibrinogen receptor). Its hidden epitopes become exposed on the platelet membrane after reacting with calcium-chelating agents commonly used as anticoagulants in whole blood sampling tubes.

Pseudothrombocytopenia is most frequently caused by EDTA, but it can be associated with the presence of sodium citrate and lithium heparin, as well as other anticoagulants (*2*, *3*, *5*). Once these anticoagulants come in contact with the whole blood in which anticoagulant-dependent antibodies with optimal activity at temperatures below normal body temperature are present, platelet aggregation will occur, eventually resulting in PTCP (*8*).

According to the recommendations issued by the International Society for Laboratory Hematology (ISLH), the peripheral blood smear should be reviewed: a) if the platelet count determined by automated hematology analyzers is below 100 x10^9^/L (at first patient measurement), and b) if the delta check of the platelet count (*i.e.* the difference between the current and previous patient’s result) exceeds the criteria of 40% (*10*). Besides these quantitative criteria, the ISLH recommends review of the peripheral blood smear regardless of the platelet count if any of the following flags are generated during automated complete blood count (CBC) analysis: the presence of platelet clumps, giant platelets or any other flag related to abnormalities in platelet distribution and/or size (*9*, *10*).

Available automated methods can provide reliable platelet counts in the presence of large platelets and fragments of other blood cells; however none can accurately determine the platelet count when aggregates are present in the sample. Importantly, the generation of flags related to platelet aggregation depends on the analyzer settings and analytical technique used (Table 1). Notably, the impedance method is the most sensitive to the presence of platelet aggregates (*2*). Therefore, reviewing the platelet histogram obtained by impedance method is considered an important step in raising suspicion of PTCP, regardless of platelet count obtained or flag presence. Atypical platelet histograms, displaying sawtooth features and serrated curve tails without baseline approaching at 20 fL, might indicate platelet aggregation (Figure 1). White blood cell fragments, red blood cell fragments and microcytes might also interfere with automated platelet counting (*2*, *3*).

**Table 1:** Overview of the most common hematology analyzers in Croatia with corresponding flags for suspecting PTCP

**Manufacturer**	**Analyzers**	**Instrument flags**
Sysmex (Sysmex, Kobe, Japan)	XS, XE, XN, XT, XP, KX series, pocH 100i	PLT clumps Plt Abn Distribution Giant Platelets
Beckman Coulter (Beckman Coulter, Miami, USA)	ACT, UniCel DxH, LH750, HmX	Platelet Clumps Giant Platelet PLT Inter: Debris RBC-PLT Overlap
Siemens (Siemens, Marburg, Germany)	Advia 360, 120, 560, 2120i	PLT-CLM Plt Clumps Large PLT
Mindray (Shenzhen Mindray Bio-Medical Electronics CO., Ltd., Shenzhen, China)	BC 5150, BC 5380, BC 5390	Plt Clump?
Abbott (Abbott, Santa Clara, USA)	Alinity hq, Cell-Dyn 1800, 3700, Ruby, Emerald	PLT Clump No MPV result NRBC NWBC
PTCP - pseudothrombocytopenia. PLT - platelet count. RBC - red blood cell count. MPV - mean platelet volume. WBC - white blood cell count.		

## Recommended laboratory procedure for the detection and management of EDTA-induced PCTP

**Figure 2:**
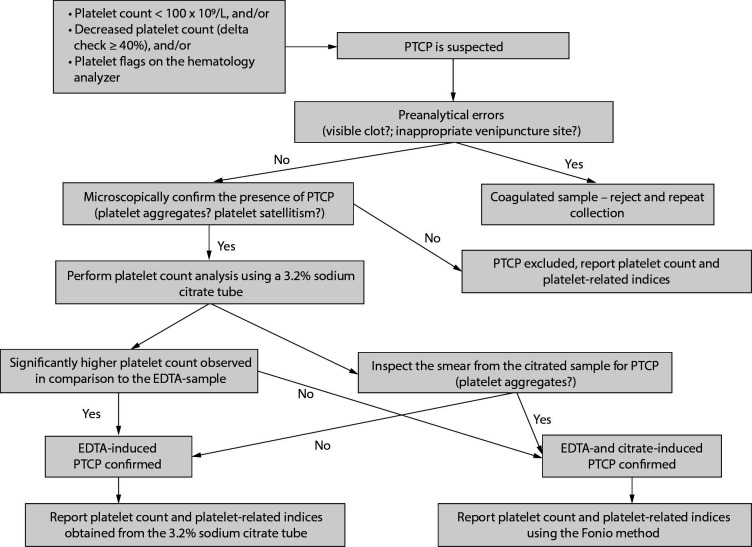
Steps in the management of suspected EDTA-induced pseudothrombocytopenia. PTCP - pseudothrombocytopenia.

The recommended procedure for detection and reporting platelet count when PCTP is suspected is described below. The procedure is summarized in Figure 2.

### 1. Exclusion of the influence of preanalytical errors

Inadequate preanalytical sample handling (*i.e.* tube filling or blood mixing after collection) might lead to falsely decreased platelet count. If PTCP is suspected according to the previous criteria, the following should be excluded:

1. *in vitro* activation of the coagulation process, either by detecting a visible clot or a fibrin strand in the EDTA-sample. Such samples should be rejected and sample collection should be repeated.
2. Selection of inappropriate venipuncture site – sample collection near the infusion line can lead to contamination of the collected sample with the fluids being infused and, consequently falsely lowering all cell counts, including the platelet count. Whenever possible, an alternative blood collection site, preferably from the opposite arm, should be selected to avoid contamination (*12*).

After exclusion of the influence of preanalytical factors on the platelet count, a reflex testing for platelet count analysis might be performed using an alternative analytical method (optical or fluorescence platelet counting), if available. The result obtained for platelet count analysis using the alternative method should be regarded as informative, not definitive (*13*, *14*).

### 2. Microscopic confirmation of PTCP

**Figure 3:**
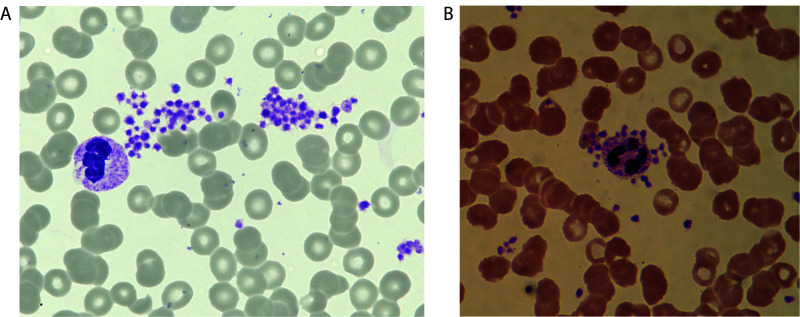
(A) Platelet aggregates and (B) platelet satelitism in peripheral blood smear prepared from EDTA-anticoagulated blood (*16*).

Once preanalytical factors are excluded, PTCP should be confirmed by morphologic assessment of the peripheral blood smear. Slide preparation and staining might be performed manually (stained with May–Grünwald Giemsa, MGG) or using an automated slide maker and stainer. The presence of platelet aggregates or platelet satellitism in EDTA-anticoagulated samples, as shown in Figure 3, should be confirmed by inspection of the peripheral blood smear using light microscopy which is the method of choice for this purpose, as recommended by the International Council for Standardization in Hematology (*15*).

After confirmation of platelet aggregates in the sample, platelet count and associated platelet parameters should not be reported. The laboratory report should clearly indicate the suspected presence of platelet aggregates confirmed by microscopic examination (Figure 4).

### 3. Measuring platelet count in samples with suspected EDTA-induced PTCP

In samples with microscopically confirmed PTCP, platelet count and platelet-related indices (mean platelet volume (MPV), plateletcrit (PCT), and others) should not be reported.

The responsible medical biochemist/specialist in medical biochemistry and laboratory medicine should directly contact the patient’s healthcare provider, comment the findings and request a repeated sample collection using a 3.2% sodium citrate tube for platelet count determination. If the patient’s healthcare provider cannot be reached, the patient should be directly invited for repeated blood sampling.

In cases when a corresponding 3.2% sodium citrate tube is already available in the laboratory (*e.g.* when coagulation testing has been requested), it should be used for platelet count determination when EDTA-induced PTCP is suspected, without the need for repeated blood sampling.

Platelet count should be determined on the hematology analyzer from the 3.2% sodium citrate tube and the result should be multiplied by 1.1 to eliminate the dilution effect of the liquid citrate anticoagulant.

The platelet count and platelet-related indices are reported from the 3.2% sodium citrate tube after confirmation that the PTCP is EDTA-induced, that is in cases when:

1. a significantly higher platelet count is observed in comparison to the EDTA-sample platelet count, or
2. the presence of platelet aggregates in the 3.2% sodium citrate tube is excluded after microscopic evaluation of the peripheral blood smear prepared from the 3.2% sodium citrate tube.

If the platelet count measured from the 3.2% sodium citrate tube is not significantly higher or platelet aggregates are present in smears prepared from the 3.2% sodium citrated sample (the incidence is in about 10-20% of patients), both EDTA- and citrate-induced PTCP are confirmed. In this case platelet counts might be determined using the Fonio manual counting method (see Appendix 1) (*17*).

A „significantly higher“ platelet count (*i.e.* the exact rise in platelet count which should be obtained from the citrated sample to confirm EDTA-induced PTCP) cannot be unequivocally and strictly defined because it depends on multiple factors (EDTA-sample transport time and temperature, number and morphology of platelet aggregates present in the EDTA-sample, original platelet count, patient’s medical history, *etc.*). Thus, the quantification of a „significantly higher“ platelet count determined from the 3.2% sodium citrate tube is individual and depends on the responsible medical biochemist/specialist in medical biochemistry and laboratory medicine.

### 4. Reporting platelet counts

If EDTA-induced PTCP is suspected due to the presence of platelet aggregates in the peripheral blood smear, the platelet count and related platelet indices (MPV, PCT, *etc.*) should not be reported. All other results pertaining to the complete blood count (CBC), should be reported as determined from the EDTA-sample on the hematology analyzer. A comment should be clearly indicated on the corresponding laboratory report (Figure 4A): *Due to suspected EDTA-induced pseudothrombocytopenia in the sample, blood sampling in a tube with sodium citrate as the anticoagulant is required.*

**Figure 4A:**
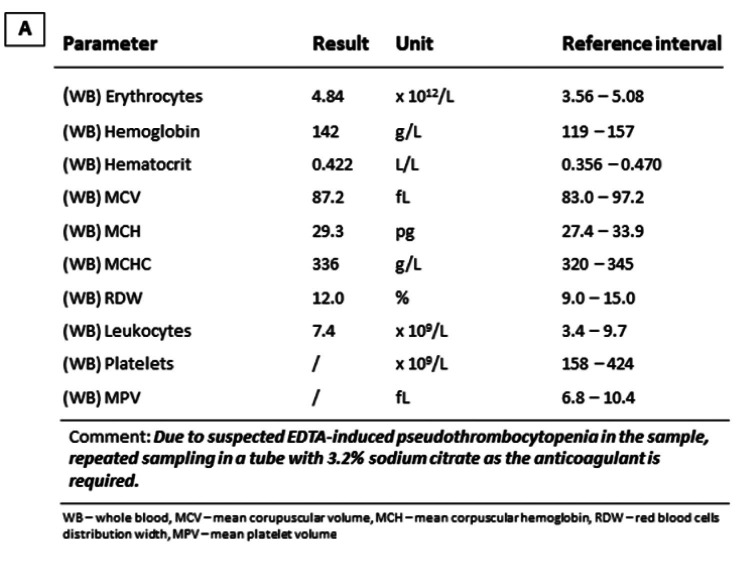
Examples of laboratory reports for the following situations: (A) platelet aggregates are found in the EDTA-sample and EDTA-induced pseudothrombocytopenia is suspected, (B) reporting of platelet count and platelet-associated parameters from the sodium citrate tube, (C) reporting of platelet count and platelet-associated parameters from the sodium citrate tube if these parameters are available as separate requests from the laboratory information system, (D) platelet aggregates are found both in the EDTA and citrate sample and (E) reporting of the platelet count using the manual Fonio counting method. Reference intervals are derived from the Croatian national harmonisation project of reference intervals (*18*).

If platelet clumps are not encountered in the citrated sample, the platelet count and related platelet indices should be reported as determined by the hematology analyzer from the 3.2% sodium citrate tube and this should be clearly indicated on the report as shown on Figure 4B. Additionally, if analyses from the 3.2% sodium citrate tube are available as separate single requests from the laboratory information system, platelet count and related platelet indices should be reported without the need for commenting (Figure 4C).

**Figure 4B:**
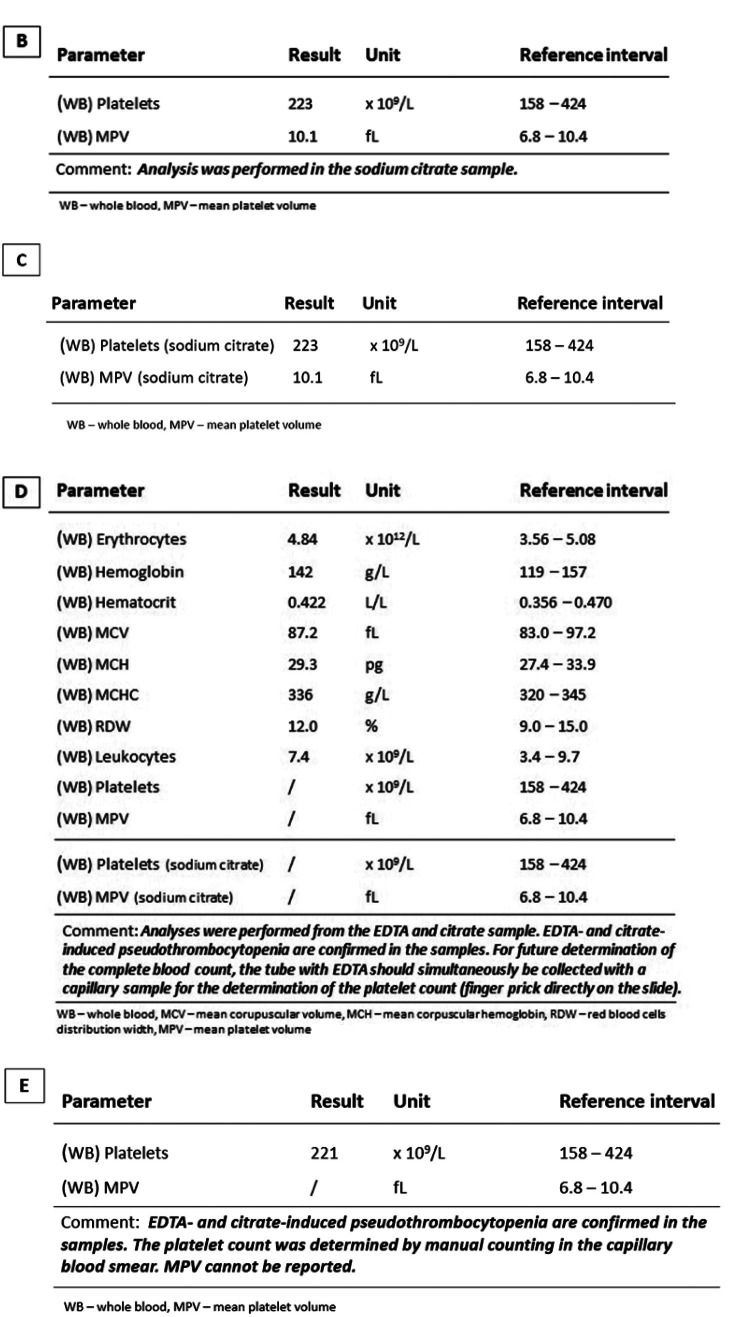
Continued.

If PTCP with both types of anticoagulants (EDTA and 3.2% sodium citrate) has been confirmed, the platelet count and related platelet indices (MPV, PCT, *etc.*) should not be reported. All other results pertaining to CBC should be reported as determined from the EDTA-sample on the hematology analyzer. The platelet count should be reported as determined by the Fonio manual counting method from a capillary sample. A comment should be clearly indicated on the corresponding laboratory report: *Analyses were performed from the EDTA and citrate sample*. *EDTA- and citrate-induced pseudothrombocytopenia are confirmed in the samples. For future determination of the complete blood count, the tube with EDTA should simultaneously be collected with a capillary sample for the determination of the platelet count (finger prick directly on the slide)* (Figure 4D).

When platelet count is determined in a capillary sample using the Fonio manual counting method, MPV should not be reported and an appropriate comment should be included within the laboratory report: *EDTA- and citrate-induced pseudothrombocytopenia are confirmed in the samples. The platelet count was determined by manual counting in the capillary blood smear. MPV cannot be reported* (Figure 4E).

### 5. Reporting platelet count from samples with excluded PTCP

If the presence of EDTA- and/or citrate-induced PTCP is excluded, the platelet count determined on the hematology analyzer from EDTA-sample should be reported. A comment should be indicated on the laboratory report: *The obtained results exclude the presence of EDTA-induced pseudothrombocytopenia.*

## Data Availability

No data was generated during this study.

## Appendix 1 Platelet counting method according to Fonio

### Method principle

Platelet count according to Fonio is a manual counting method for determining the number of platelets and is used only if platelet count cannot be reliably determined by automated counting methods on a hematology analyzer (*e.g.* if platelet aggregates are still present in sample with 3.2% sodium citrate) or when the unreliability of platelet counts obtained by automated analyzers is suspected (*e.g.* in the presence of macroplatelets, an unusual histogram or scatter diagram and other individual cases based on the laboratory professional’s assessment) (*17*).

If EDTA- and citrate PTCP is confirmed, platelets might be counted according to Fonio by collecting finger stick capillary blood, preparing a stained smear using the MGG method, and counting platelets *per* thousand erythrocytes under a light microscope. If aggregates are not present, but platelets still need to be counted according to Fonio, a smear can be made directly from a test tube without the need for transferring the blood drop directly from the finger to the slide.

### Procedure

The fingertip is disinfected and pricked with a lancet. The resulting drop of blood is transferred directly from the fingertip to the microscopic slide by placing the upper surface of the slide in contact with the drop of blood. After that, a smear is prepared using the MGG (Pappenheim) staining method. Platelets are counted under the immersion lens of a light microscope at 100x magnification. It is necessary to count platelets alongside with erythrocytes, until the number of a thousand erythrocytes is achieved (usually 5 fields with 200 erythrocytes in each). The platelet count is obtained by multiplying the erythrocyte number determined on the automated analyzer and the number of platelets counted in the smear:

Platelet count (x10^9^/L) = A x Erc / 1000

where A is the number of platelets counted *per* 1000 erythrocytes and Erc is the number of erythrocytes determined on the analyzer (x10^12^/L).
